# Foliar application of green-synthesized Cu–Zn nanocomposites: improve physiological responses, isozymes activity, and photosynthetic traits in lead-stressed pea (*Pisum sativum* L.) plants

**DOI:** 10.1038/s41598-026-43558-w

**Published:** 2026-03-26

**Authors:** Mahmoud S. Osman, Salem S. Salem, Hossam M. Fouda, Amr H. Hashem, Sahar I. ELshennawy, Eman N. Mustafa, Eman G. El-Hosary

**Affiliations:** 1 Botany and Microbiology Department, Faculty of Science, Al-Azhar University, Cairo11884, Nasr City, Egypt; 2 Botany and Microbiology Department, Faculty of Science, Girl’s Branch, Al-Azhar University, Cairo, Egypt; 3 Botany and Microbiology Department, Faculty of Science, Damanhour University, Damanhour, Egypt

**Keywords:** Heavy metals, Oxidative stress, Isozymes, Lead acetate, Osmolytes, Protein pattern, Biochemistry, Environmental sciences, Physiology, Plant sciences

## Abstract

Heavy metal contamination of the soil has grown in importance in recent years. It may reduce agricultural output and jeopardize the welfare of individuals by entering the food chain. The use of nanomaterials, such as copper-zinc (Cu-Zn) nanocomposites, has emerged as a potential strategy to alleviate lead (Pb) stress in plants. Chlorophyll content, biomass accumulation, root and shoot length, and other aspects of plant development were used to assess the impacts of these nanomaterials. Additionally, protein patterns and antioxidant isozyme analysis, including Antioxidant enzymes, were assessed to understand the mechanisms underlying the plant’s response to Cu-Zn exposure. The results suggest that after harvesting of the 35-day-old pea plant, signs of the plant’s amelioration from stress destruction included morphological traits, photosynthetic pigments, sugars, phenol content, protein levels, Pb accumulation, protein pattern, and isozymes analysis were performed. In stressed plants, measurements of growth parameters, physiological aspects, all showed severe declines. The study discovered that pea plants’ growth indices, chlorophyll content, and osmolytes were all improved by using foliar application of green-synthesis copper zinc (Cu-Zn) nanocomposites. Additionally, it lessened the negative effects of lead (Pb) stress on pea plants. Thus, Cu-Zn nanocomposites may be a viable approach to reducing heavy metal stress and enhancing pea plant crop growth and output.

**Supplementary Information:**

The online version contains supplementary material available at 10.1038/s41598-026-43558-w.

## Introduction

High or low temperatures, waterlogging, drought, salinity, heavy metals (HMs), and ultraviolet (UV) radiation are examples of abiotic stressors that can impact modern agricultural productivity globally^1,2^. The way plants respond to abiotic stress varies depending on the crop, stress, and exposure time^3,4^. With the world’s population continuing to expand, abiotic stressors—which modify plant cells physiologically and biochemically and can have detrimental effects on plant growth, development, and productivity—are having a greater influence on food production^5,6^. Researchers are working to improve crop yield and food production, alleviate the adverse consequences of abiotic stressors, and attain sustainability and food security to satisfy the world’s expanding food demand. To overcome these pressing global issues, researchers must keep creating novel technologies or solutions^7,8^.

Elements that are necessary for plant growth and development include Co, Cu, Fe, Mn, Mo, Ni, and Zn; non-essential elements include Cd, Cr, Pb, and mercury. All HMs, however, are extremely harmful to plants when present in high quantities^9,10^. Many metabolic systems are negatively impacted by HMs at toxic doses. Among these are the following: suppression of photosynthesis, respiration, and enzymatic activities^7^ and displacement of protein structures brought on by the formation of bonds between the HMs and sulfhydryl groups^11,12^.

Heavy metals have surpassed insecticides and well-known pollutants like sulfur dioxide and carbon dioxide in recent decades^13^. According to^14^, 70% of all heavy metals and their compounds that are transported through the human body originate from food. Numerous sources, including human activity, emit heavy metals (HMs) into the air, soil, and water^15,16^. Metal poisoning increases the danger and creates issues for both people and animals since they are later transferred into the food chain^17^. One significant problem that impairs crop productivity and jeopardizes food security is heavy metal contamination.

The use of nanoparticles (NPs) to reduce Lead toxicity is new, effective, and promising. NPs strengthen the plant antioxidant system, enhance the apoplastic barrier, reduce the mobility of lead (Pb) in soil, and boost the synthesis of secondary metabolites, especially phenols. In addition to their mimetic properties, these NPs can boost the synthesis of secondary metabolites such as phenolics, which are powerful stress relievers in plant physiology. This method presents a viable substitute for traditional plant growth regulators^18,19^. Another more effective method of increasing plant resistance to HM is foliar spraying, which is applied outside of the soil^20^. While Ba (barium), Sb (antimony), Mo (molybdenum), Tl (thallium), and Sn (tin) are regarded as less harmful than others, heavy metals (HM) are regarded as extremely dangerous^21^.

The positive applications of nanotechnology in improving crop quality and productivity are well known. Among these uses are its ability to lessen the effects of climate change and abiotic stresses^22,23^. To increase agricultural production and development, nano-based technologies are used, such as nano-sensors and nano-fertilizers. Combining these technologies with foliar sprays, soil irrigation, seed coatings, and genetic engineering can increase photosynthesis effectiveness and disease resistance, assess the nutritional requirements of plants, and provide several advantages over conventional fertilizers. These benefits come from their high surface-area-to-volume ratio, higher efficiency in removing pollutants, and capacity to effectively supply essential nutrients to plants and soil as nanofertilizers^24^.

Several investigations have shown that nanomaterials improve plant adaptability and increase crop yields in response to abiotic stress, specifically Lead toxicity^25,26^. They do this by supplying essential nutrients and lowering nutrient losses because of their large surface area and nutrient-holding capacity^2^, reducing lead absorption, increasing antioxidant defense, enhancing the efficiency of photosynthesis, and regulating botanical hormone levels. Their physical (carbon-based NPs with small pores that may absorb and retain soil micronutrients) and chemical (NPK-NPs) characteristics are linked to their nutrient-holding capacity^27^.

The green synthesis process has been acknowledged as a viable option to overcome the drawbacks of chemical processes. During the preparation process, this approach applies plants, certain fungi, and microorganisms^28–33^.

*Pisum sativum*, the garden pea, is a member of the pea family, Fabaceae. The genus *Pisum* has several species, including *Pisum sativum*. A significant legume crop, *Pisum sativum* L., is sometimes referred to as green pea, dry pea, or field pea. It is a strong source of protein, vitamins, minerals, and bioactive substances that are good for human health^34,35^. Nearly every nation in the globe grows peas, which are considered a vital component of the human diet^36^. As a result, peas are high in nutrients and bioactive substances and have a promising future as functional meals or health products. Peas also include other healthy substances like zeaxanthin and β-carotene. Numerous in vitro and in vivo experimental studies have shown the numerous health advantages of peas, including their anti-inflammatory, anti-hypertensive, anti-obesity, anti-cancer, anti-fatigue, anti-diabetic, antibacterial, and anti-renal fibrosis properties. Due to the presence of several bioactive components (such as polyphenols, polysaccharides, and peptides), several studies have demonstrated that peas and their byproducts have exceptional antioxidant activity^37,38^.

The current research investigation sought to develop copper-zinc nanocomposites via biological processes and determine whether they could help pea plants overcome the negative effects of heavy metal stress by tracking the plants’ physiological and biochemical processes, along with some of their nutritional attributes.

## Materials and methods

### Collection and identification of plant samples

A sufficient number of the present plants were collected from their natural habitats in Marsa Matruh, Egypt, at Latitude: 31.3543° N, Longitude: 27.2373° E during August 2023. The plants collected under the IUCN Policy and identified by Dr Sahar I. ELshennawy, lecturer of Plant Ecology, Faculty of Science, Al-Azhar University, Cairo, Egypt. A voucher specimen was deposited at the herbarium of the Botany and Microbiology Department, Faculty of Science, Al-Azhar University, Cairo, Egypt. The permissions for the collection of plants were obtained for scientific research purposes. The plant was identified by (Täckholm 1974; Boulos 1999) and was recognized as *Cakile maritima*.

### Preparation of biological Cu/Zn nanocomposite

A green co-precipitation method was utilized to synthesize Cu/Zn nanocomposites in a single step, employing plant extract of *Cakile maritima* Scop as a natural reducing and capping agent (5gm plant powder per 100 ml distilled water). In this procedure, 1 g of copper (II) acetate monohydrate and 1 g of zinc acetate dihydrate were each dissolved in 20 mL of distilled water. The metal precursor solutions were then slowly added to 80 mL of an aqueous plant extract, which was then heated for three hours at 70 °C while being stirred magnetically. The pH of the reaction mixture was carefully adjusted to 10 by the dropwise addition of sodium hydroxide solution while maintaining constant stirring. A visible precipitate was observed during the reaction, indicating the formation of the Cu/Zn nanocomposites. The resulting solid was separated by centrifugation, thoroughly washed with distilled water to remove residual impurities, and subsequently dried in a hot air oven at 80 °C for 48 h.

### Characterization of Cu/Zn nanocomposite

FTIR spectra of Cu/Zn nanocomposite functional groups were obtained using Nicolet iS50, Thermo Scientific, USA, of resolution 4 cm⁻¹ and with 64 cumulative scans in the 400–4000 cm⁻¹ range. KBr pellets of the samples were prepared before analysis. Morphological features and fracture morphology of the nanohybrids were examined by transmission electron microscopy (TEM; JEOL JEM-2100, Japan) at 200 kV accelerating voltage. Crystallographic characteristics were analyzed by X-ray diffraction (XRD) on a Bruker D8 Advance Diffractometer using Cu-Kα radiation (λ = 1.54 Å, 40 kV, 30 mA). A field-emitted electron microscope (TEM) fitted with a Field Emission-Gun and linked to an energy-dispersive X-ray analyzer with a 30 kV stimulation source for energy-dispersive X-ray investigation (EDX) and mapping was used to analyse the surfaces of the prepared Cu/Zn nanocomposite.

### Treatment and growing conditions

The Agricultural Research Centre in Giza, Egypt, generously provided the pea seeds (*Pisum sativum*, cv. Master-B). The seeds were soaked in 4% sodium hypochlorite for two minutes to surface sterilize them before germination, and they were then repeatedly cleaned with distilled water. Climate indicators included: an average day/night temperature cycle of 25/14, light 10/14 h, and air humidity between 50% and 70%. The pots were separated randomly with three replication into six sets as follows: − 1- Control (irrigated with fresh water (FrW), 2- Cu/Zn nanocomposite (50 mg L^− 1^), 3- Cu/Zn nanocomposite (100 mg L^− 1^), 4- Control heavy metal HM (irrigated with 100 ppm Lead acetate), 5- Cu/Zn nanocomposite (50 + HM), and 6- Cu/Zn nanocomposite (100 + HM). The nanocomposite Cu/Zn nanocomposite was foliar applied 3 times (10, 20, and 30 days after sowing 1 time each week with a range of (300 mL/pot). While the control plants were foliar applied with tap water. The using of lead acetate concentration 100 ppm was according to previous studies^39^.

Throughout the whole study period, irrigation was done to the plants whenever required. Plants that were 35 days old were taken, cleaned with tap water and then distilled water to get rid of dirt particles, and then gently blotted with tissue paper. The plants were then dissected to remove the roots and shoots and promptly stored for the measurement of several growth biomarkers. Other samples were preserved for chemical analysis as fresh or dried in an oven at 60 °C to a consistent weight.

### Photosynthetic pigment determination

1 gram of fresh leaves was grinded with a mortar and pestle in 100 milliliters of 80% aqueous acetone (v/v), filtered by filter paper Whatman Grade 1, and then the volume was increased to 100 milliliters using 80% acetone per Vernon and Seely^40^ methodology for determining pigments.

### Determination of the content of osmolytes

The amount of total soluble sugars was assessed according to the technique outlined by^41^. To extract soluble sugars, dried shoots (0.5 g) were combined with 2.5 mL of 2% phenol and 5 mL of 30% trichloroacetic acid, then filtered via filter paper. 1 mL of the filtered solution was then mixed with 2 mL of the anthrone reagent (2 g anthrone/L in 95% H_2_SO_4_). The produced blue-green color was measured at 620 nm. As per Lowry et al.^42^, the soluble protein content of the dry shoot was calculated by adding 1 g of the dried leaves to 5 ml of 2% phenol water, which was then shaken and left overnight to filter the entire volume to 50 ml with distilled water.

### Determination of total phenol content

The total phenol content was determined using the procedure of Dai et al.^43^. In this technique, 1 g of dry pea shoots was extracted in 5–10 mL of 80% ethanol for at least 24 h. After explaining alcohol, the leftover residue was extracted three times using five to ten milliliters of 80% ethanol. After that, 50 milliliters of 80% ethanol were added to the cleared extract. After thoroughly mixing 0.5 mL of the extract with 0.5 mL of Folin’s reagent, the entire mixture was shaken for three minutes. Three milliliters of distilled water and one milliliter of saturated Na_2_CO_3_ solution were added, and everything was thoroughly mixed. The blue color was measured at 725 nm after one hour, and gallic acid was employed as the standard compound for calibration.

### Estimation of hydrogen peroxide (H_2_O_2_) and malondialdehyde (MDA) contents

The method described by Mukherjee and Choudhuri^44^ was carried out for the estimation of H_2_O_2_ content. Where fresh specimens (0.05 g) were extracted with 4 mL of cold acetone. An aliquot (3 mL) of the extracted solution was combined with 1 mL of 0.1% titanium dioxide in 20% (v: v) H_2_SO_4,_ and the combination was then centrifuged at 6000 rpm for 15 min. At 415 nm, the supernatant’s yellow color intensity was assessed. The MDA content was estimated according to the method of Heath and Packer^45^ and the MDA concentration was calculated using its extinction coefficient 155 mM^−1^cm^− 1^. In this procedure, fresh leaf samples (0.5 g) were extracted with 5% trichloroacetic acid and centrifugated at 4000 g for 10 min. Then, 2 mL of the extract were combined with 2 mL of 0.6% Thiobarbituric acid (TBA) solution. Following this, the resulting mixture was put in a water bath for 10 min. Following cooling, the resulting color’s absorbance was measured at 532, 600, and 450 nm.

### Determination of Pb content

The content of Pb in roots and shoots were determined as the standard method described by Kimbrough and Wakakuwa (1989) using Inductively Coupled Plasma–Optical Emission Spectroscopy (ICP–OES). The samples were placed in a beaker and then 3 ml of HNO_3_ was added and stood for 10–20 min, then heated at 90–95 c for 90 min until the volume reduced about 1–2 hr. Then, cool, and 1 ml of H_2_O_2_ was added and reheated again for 20–30 min. until the solution is clear. After digestion, cool at room temperature, and evaporate to near dryness, then the residue is dissolved in 10 ml of 1% HNO_3,_ followed by filtration and then transferred to acid washed volumetric flask and brought to volume with deionized water (1% HNO_3_ final). The R^2^ was typically ≥ 0.995, LOQ fell in roughly 0.1-1.0 ugL^−1^Caculated as (10 × SD (acid + H₂O₂, no sample as blank).

### Protein patterns

Fresh pea leaves were extracted by crushing the appropriate amount of each sample (0.25 g) with an extraction buffer and shaking. Following their placement in Eppendorf tubes, the extracts were centrifuged for 10 min at 1000 rpm while being cooled. Following the procedures outlined by Laemmli^46^ and modified by Studier^47^, protein extraction was carried out as demonstrated by^48^. The molecular weight of the proteins was then determined with the marker, a protein with a broad range of molecular weights (Gene Direx.com).

### Isozymes electrophoresis

The procedure was used to evaluate the peroxidase (POD) isozyme Thipyapong et al.^49^. The isozyme polyphenol oxidase (PPO) was calculated according to the Barceló et al.^50^ approach. The relative distance (Rf-value) of the bands on the gel was calculated as described by Manganaris and Alston^51^ using rf = 1.0, distance to the fastest band, and Rf = 0.0, the starting point.

### Statistical analysis

Using the resultant data, one-way variance analysis (ANOVA) was performed. Statistically significant changes between treatments were shown at *p* < 0.05 using the least significant difference (LSD test) using CoStat (CoHort, Monterey, CA, USA). Mean ± standard deviation are displayed for a sample size of three replications.

## Experimental results

### Fourier transform infrared spectroscopy (FTIR) analysis

**Fig. 1:**
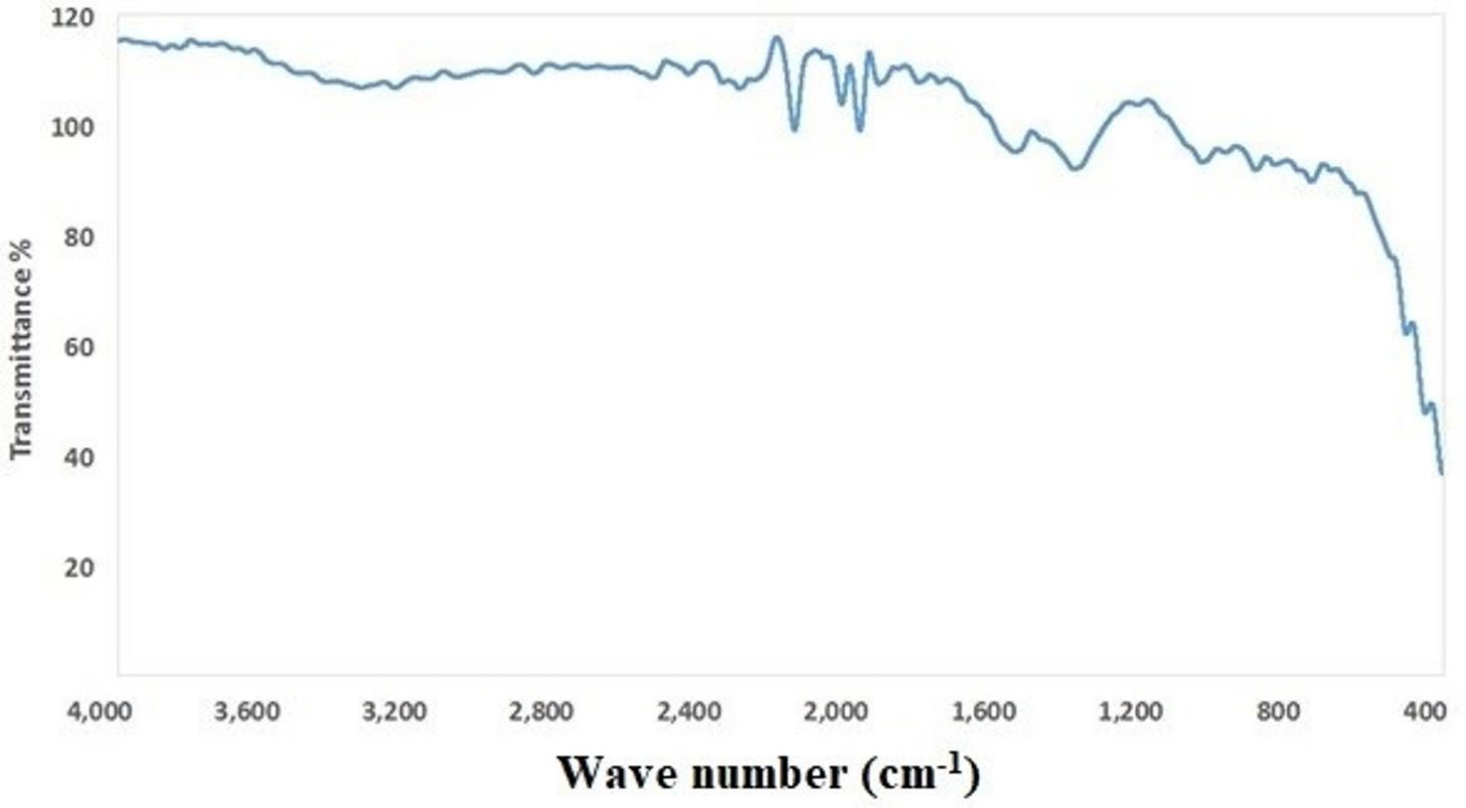
FTIR spectrum of Cu/Zn nanocomposite.

FTIR spectroscopy as shown in Fig. 1, was employed to identify the functional groups involved in the reduction and stabilization of Cu/Zn nanocomposite. The FTIR spectrum of Cu/Zn nanocomposite exhibited characteristic absorption bands at 3244.9 cm⁻¹, 2411.7 cm⁻¹, 2164.2 cm⁻¹, 1987.2 cm⁻¹, 1561 cm⁻¹, 1399.8 cm⁻¹, 1053.7 cm⁻¹, 760.3 cm⁻¹, 508 cm⁻¹, and 465 cm⁻¹, indicating the presence of phenolic compounds, alcohols, alkanes, and proteins. These bands indicate their role in the formation of Cu/Zn nanocomposite and surface capping.

### X-Ray Diffraction (XRD)

**Fig. 2:**
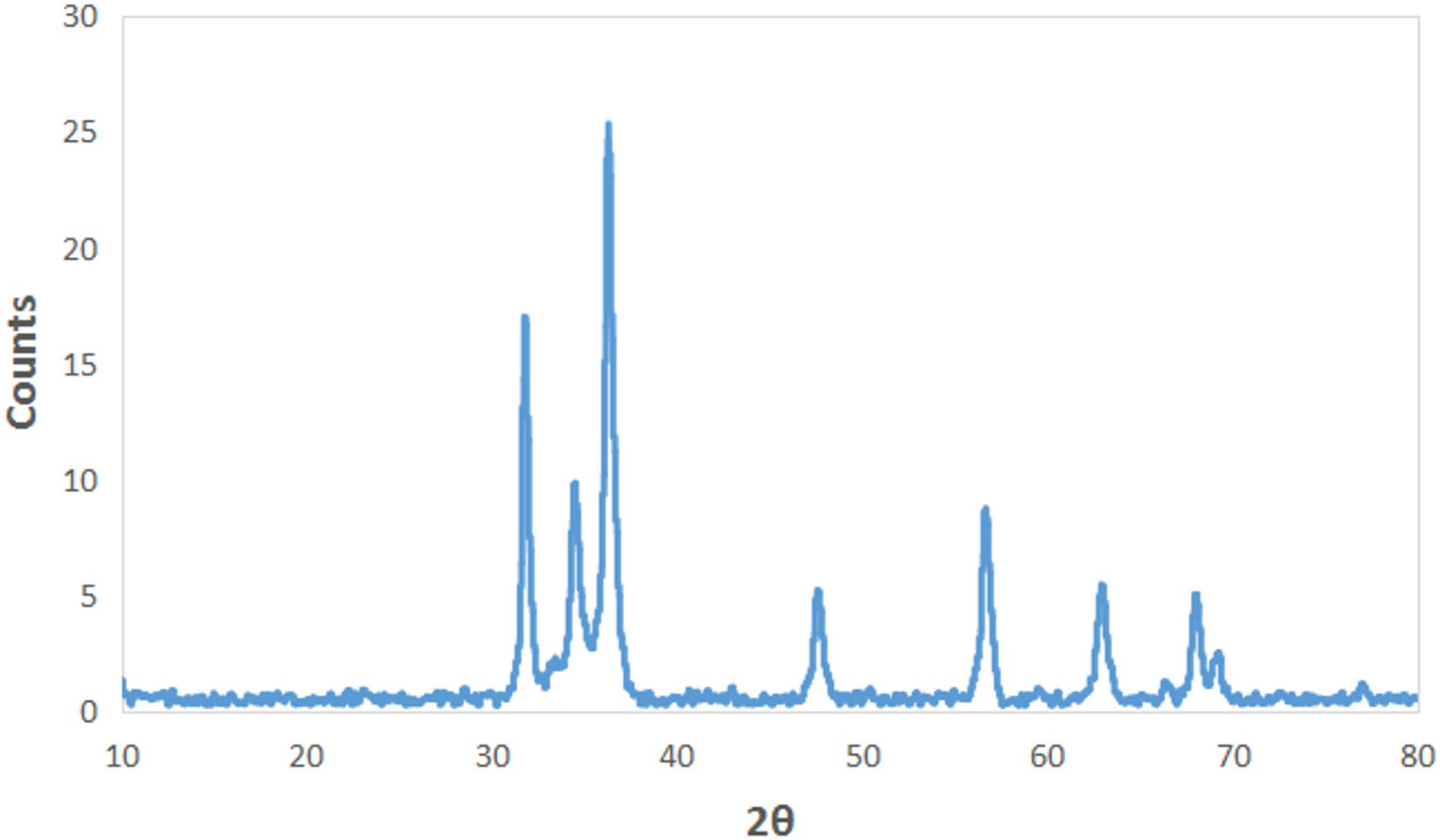
XRD of Cu/Zn nanocomposite.

The structural characteristics of the plant extract-mediated Cu/Zn nanocomposite were investigated using X-ray diffraction, as depicted in Fig. 2. The diffractogram displays well-defined diffraction peaks positioned at approximately 2θ = 31.9°, 34.2°, 36.4°, 47.9°, 56.9°, 62.8°, and 67.9°, indicating the formation of crystalline domains within the synthesized nanocomposite. The prominent peak at higher angles reflects a highly crystalline phase, while the broader reflections at lower angles suggest the presence of nanocrystalline or partially amorphous regions, likely influenced by the reducing and stabilizing action of metabolites in plant extract.

### TEM and EDX analysis

**Fig. 3:**
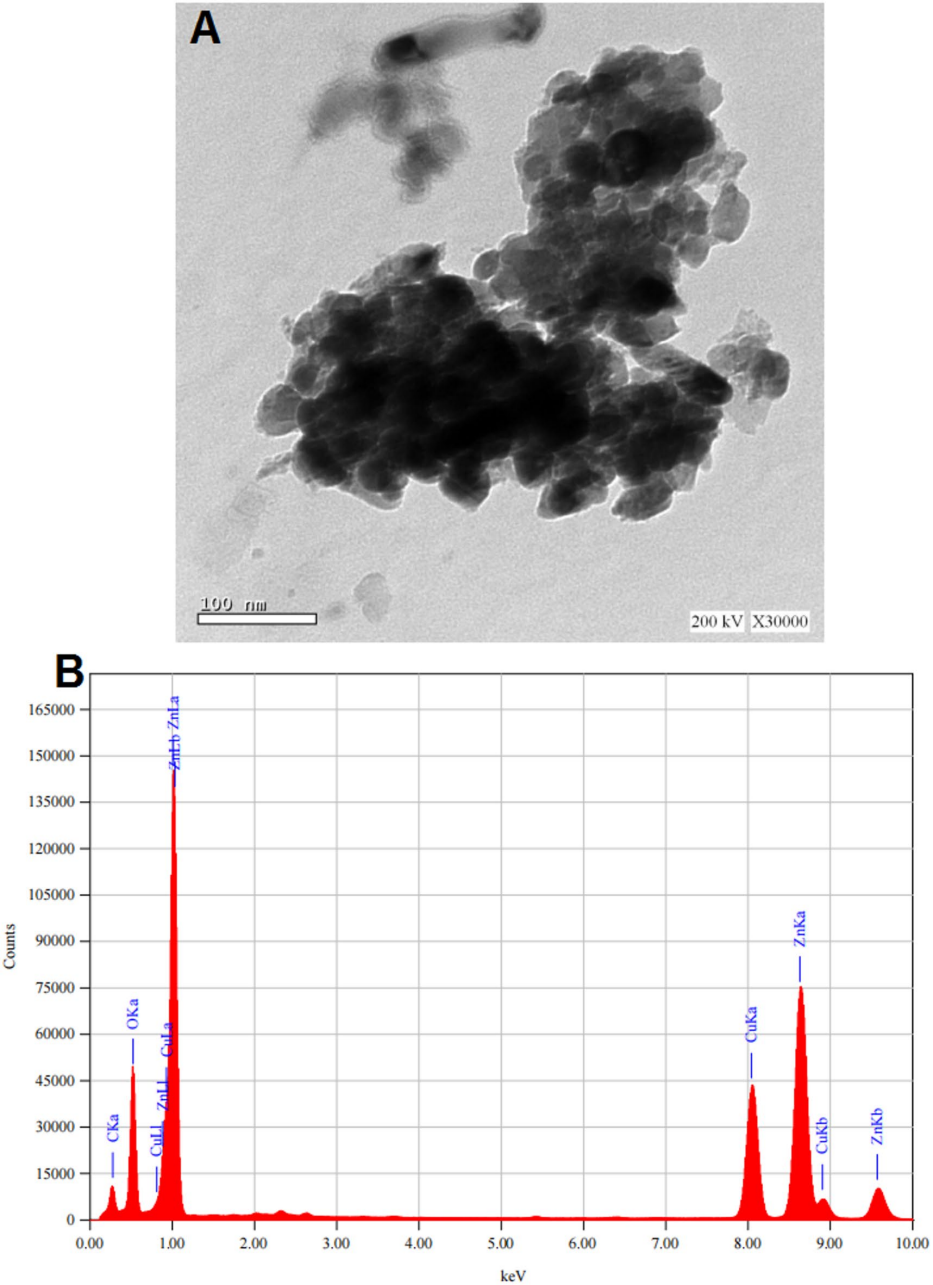
TEM image (**A**) and EDX (**B**) spectrum of Cu/Zn nanocomposite.

Transmission Electron Microscopy (TEM) analysis (Fig. 3A) demonstrated that the CuZn nanocomposites possess a nearly spherical morphology with slight aggregation. Individual particles were measured directly from the micrograph and found to range between 25 and 70 nm in diameter. This morphology indicates successful nanoparticle synthesis and suggests effective stabilization throughout the fabrication process. Complementary energy-dispersive X-ray spectroscopy (EDX) analysis (Fig. 3B) verified the elemental composition of the nanocomposites, detecting carbon{C}, oxygen{O}, copper{Cu}, and zinc{Zn}. The predominance of zinc and copper, together constituting the majority of the total mass, confirms their major incorporation into the nanocomposite framework.

### Photosynthetic Pigments

The observed results in Table 1 reveal that chlorophyll a, chlorophyll b, total (a + b), and carotenoid contents were highly significantly decreased in pea plants treated with heavy metals by 17.35%, 30.26%, 22.91%, and 25.78%, respectively, compared to the untreated control (FW). Results in Table 1 indicate that the application of biological Cu/Zn nanocomposite resulted in a significant increase in photosynthetic pigments in both control pea plants (untreated with heavy metals) and pea plants treated with heavy metals. Moreover, the mean results in Table 1 make it evident that, compared to the untreated control (FW), pea plants treated with heavy metals had considerably reduced levels of carotenoids and chlorophyll (a, b, and overall pigments).

**Table 1:** Shows the impact of biological composite nanoparticles on the photosynthetic pigments (mg g^− 1^ FW) and their constituent parts in pea plant leaves under normal (Fresh water (FrW)) and heavy metal stress conditions (HM). Three replicates’ means are used to determine the values. Data represents as Means ± SD. Significantly different LSD (*P* < 0.05) values are denoted by various letters in the data. FW= Fresh weight.

Treatments	Chlorophyll (a)	Chlorophyll (b)	Total (a + b)	Carotenoids
**Control (FrW)**	11.76 ± 0.30 c	8.89 ± 0.28 bc	20.65 ± 0.58 c	7.37 ± 0.65 c
**Composite Nano 50 mg L** ^**− 1**^	12.59 ± 0.52 b	9.73 ± 0.26 ab	22.32 ± 0.44 d	8.09 ± 0.47 b
**Composite Nano 100 mg L** ^**− 1**^	14.07 ± 0.31 a	10.29 ± 0.45 a	24.36 ± 0.76 a	8.91 ± 0.24 a
**(Heavy Metal HM)**	9.72 ± 0.24 e	6.20 ± 0.98 e	15.92 ± 1.13 e	5.47 ± 0.42 d
**Composite Nano 50 + HM**	10.54 ± 0.50 d	7.56 ± 0.35 d	18.10 ± 0.15 d	7.05 ± 0.060 c
**Composite Nano 100 + HM**	11.32 ± 0.44 c	8.17 ± 0.12 cd	19.49 ± 0.42 c	7.31 ± 0.20 c

### Soluble Sugars

Pea plants treated with Cu/Zn nanocomposite showed significant increases in total soluble sugar contents in both shoots and roots compared to the control (FW). (Table 2). It was observed that a higher Cu/Zn nanocomposite (composite Nano 100 FW) led to a greater increase in carbohydrate content in both shoots and roots in comparison to the control (FW).

**Table 2:** Impact of Biological Composite NPs on the total amount of soluble sugars (mg g^− 1^ DW) in pea plant shoots and roots and their constituent parts under normal (Fresh water (FrW)) and heavy metal stress conditions (HM). The values provided are the averages of three replicates. Data represents as Means ± SD. Data with substantially different LSD (*P* < 0.05) values are denoted by distinct letters. DW = Dry weight.

Treatments	Soluble Sugars (Shoot)	Soluble Sugars (Root)
Control (FrW)	26.81 ± 0.62 c	26.14 ± 0.68 c
Composite Nano 50 mg L^− 1^	28.13 ± 0.91 b	28.08 ± 0.13 b
Composite Nano 100 mg L^− 1^	33.33 ± 0.43 a	30.31 ± 0.31 a
(Heavy Metal HM)	25.39 ± 0.49 e	24.02 ± 0.43 e
Composite Nano 50 + HM	26.01 ± 0.36 de	24.97 ± 0.11 d
Composite Nano 100 + HM	26.33 ± 0.28 cd	25.96 ± 0.78 c

### Soluble Protein

Results in Table 3 showed that the application of Cu/Zn nanocomposite (composite Nano 50 FW) resulted in increase of total soluble proteins in shoot and root compared with untreated plants. At contrast total soluble proteins in shoot and root were significantly decreased in pea plants due to the treatment with heavy metals.

**Table 3:** Impact of Biological Composite NPs on the total amount of soluble protein (mg g^− 1^ DW) and its constituents in pea plant roots and shoots and their constituent parts under normal (Fresh water (FrW)) and heavy metal stress (HM) conditions. Three replicates’ means are used to determine the values. Data represents as Means ± SD. Data with substantially different LSD (*P* < 0.05) values are denoted by distinct letters. DW = Dry weight.

Treatments	Protein shoot	Protein root
Control (FrW)	21.16 ± 0.13 bc	20.40 ± 0.46 c
Composite Nano 50 mg L^− 1^	23.45 ± 0.59 a	23.05 ± 0.15 a
Composite Nano 100 mg L^− 1^	21.77 ± 0.46 b	21.91 ± 0.78 b
(Heavy Metal HM)	19.02 ± 0.20 e	18.55 ± 0.48 e
Composite Nano 50 + HM	19.91 ± 0.22 d	18.49 ± 0.89 e
Composite Nano 100 + HM	20.72 ± 0.30 c	19.28 ± 0.45 d

### Total phenol

Data in Table 4 showed that heavy metals treatment caused a significant increase in total phenol content. The increase in total phenols in the heavy metals control was 46.59% in the shoot and 34.15% in the root, compared to the normal control. It was also observed that pea plants treated with the biological Cu/Zn nanocomposite showed a significant increase in total phenols. Exposure to heavy metals and composites led to a considerable rise in the level of total phenolics. For example, treatment of pea plants with the composite Nano 100 HM increased total phenols content by 44.31% in the shoot and 33.37% in the root, compared to the control (FW).

**Table 4:** Impact of Biological Composite NPs on the total phenols (mg g^− 1^ DW) and their constituents in pea plant roots and shoots roots and shoots and their constituent parts under normal (Fresh water (FrW)) and heavy metal stress conditions. The values displayed are the averages of three replicates. Data are represented as Means ± SD. Data with substantially different LSD (*P* < 0.05) values are denoted by distinct letters. DW = Dry weight.

Treatments	Total phenolic content	
	shoot	root
Control (FrW)	0.417 ± 0.05 c	0.322 ± 0.01 d
Composite Nano 50 mg L^− 1^	0.544 ± 0.03 b	0.487 ± 0.08 c
Composite Nano 100 mg L^− 1^	0.465 ± 0.06 c	0.435 ± 0.02 c
(Heavy Metal HM)	0.895 ± 0.04 a	0.943 ± 0.02 ab
Composite Nano 50 + HM	0.935 ± 0.01 a	0.882 ± 0.03 b
Composite Nano 100 + HM	0.941 ± 0.02 a	0.965 ± 0.02 a

### Stress markers (MDA, H_2_O_2_)

**Fig. 4:**
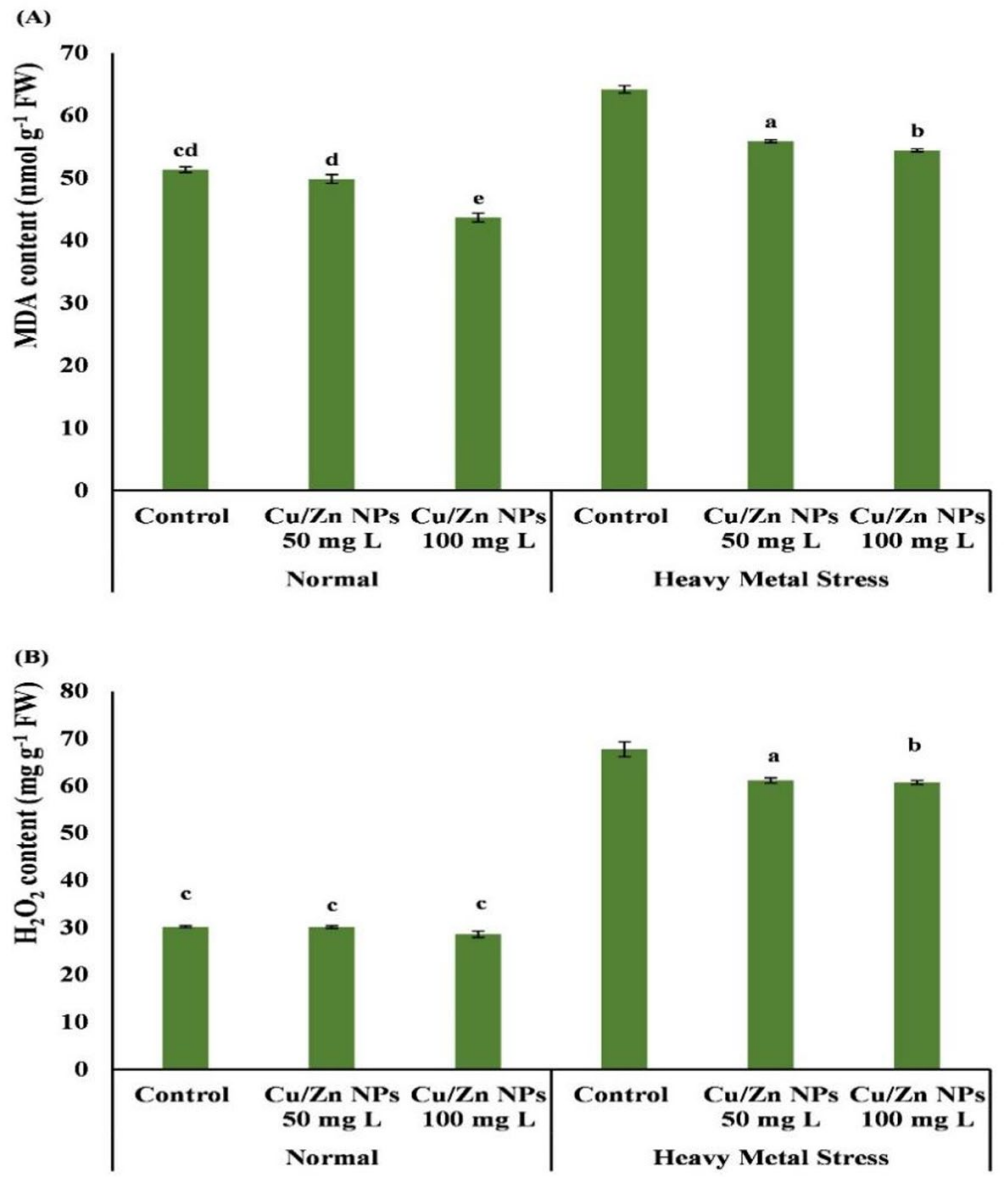
Impact of Biological Composite NPs on (a) Malondialdehyde (MDA) (nmol g^− 1^ FW), (b) Hydrogen Peroxide (H_2_O_2_) (mg g^− 1^ FW) in Pea Plant Shoots and their components under control (Fresh water (FrW)) and heavy metal stress conditions. Three replicates’ means are used to determine the values. Bars indicate Means ± SD. Significantly different LSD (*P* < 0.05) values are denoted by various letters in the data. FW= Fresh weight.

Treatment with a lower concentration of Cu/Zn nanocomposite insignificantly changed H_2_O_2_ content compared to the control (Fig. 4). Also, there was a significant decrease in H_2_O_2_ accumulation in pea plants in response to treatment with higher concentrations of Cu/Zn nanocomposite. Also, at Cu/Zn nanocomposite 50 + HM and Cu/Zn nanocomposite 100 + Hm, H_2_O_2_ content was significantly decreased by 9.73% and 10.33% respectively, of non-treated control HM (heavy metal only) (Table 5). On the contrary, there was a significant increase in H_2_O_2_ accumulation in pea plants in response to treatment with heavy metal. The increase was 44.55% compared to normal control plants. lipid peroxidation is estimated as malondialdehyde content (MDA), as the end product of lipid peroxidation. Data in Table 5 showed that treatment with composite Nano 100 FW resulted in a decrease of MDA content of 14.18% compared to control fresh water. Whereas MDA content was significantly increased in response to heavy metal concentrations (Control HM) by 1.26-fold (126.12%) compared to control (fresh water only). The MDA content in pea plants treated with Cu/Zn composite Nano 50 + HM and Cu/Zn composite Nano 100 + HM decreased by 13.4% and 14.9% of the control (HM), respectively.

### Heavy metal accumulation

Data in Table 5 showed that application of Cu/Zn nanocomposite 100 FW resulted in a considerable decrease in Pb accumulation yield by 35.71% compared to the control (fresh water only). Also, Cu/Zn nanocomposite 100 HM resulted in a significant decrease in Pb accumulation yield by 69.03% compared to the control (heavy metal only).

**Table 5:** The impact of biological composite nanoparticles on the yield of lead accumulation (mg kg^− 1^) in pea plants under normal (Fresh water (FrW)) and heavy metal stress (HM) conditions. The values provided are the averages of three replicates. Data represents as Means ± SD. Data with substantially different LSD (*P* < 0.05) values are denoted by distinct letters. *Permissible limits are according to FAO/WHO (2019, 2020).

Treatments	Pb accumulation yield
Control (FrW)	0.028 ± 0.002 d
Composite Nano 50 mg L^− 1^	0.017 ± 0.001 e
Composite Nano 100 mg L^− 1^	0.018 ± 0.0005 e
(Heavy Metal HM)	0.381 ± 0.002 a
Composite Nano 50 + HM	0.133 ± 0.002 b
Composite Nano 100 + HM	0.118 ± 0.001 c
Permissible limit*	0.20

### Isozymes analysis

**Fig. 5:**
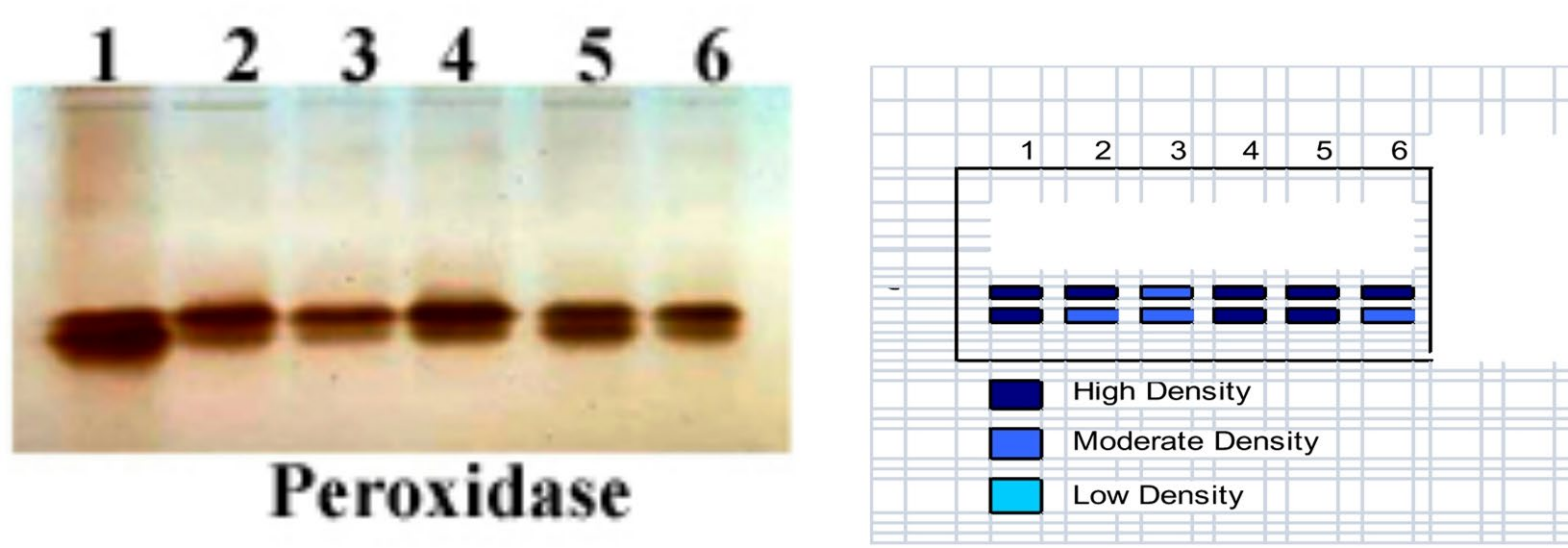
Effect of Heavy metal stress and application of biological composite nanoparticles and their interactions on (A) Peroxidase isozyme and (B) Ideogram analysis of peroxidase isozyme of pea plants.

**Fig. 6:**
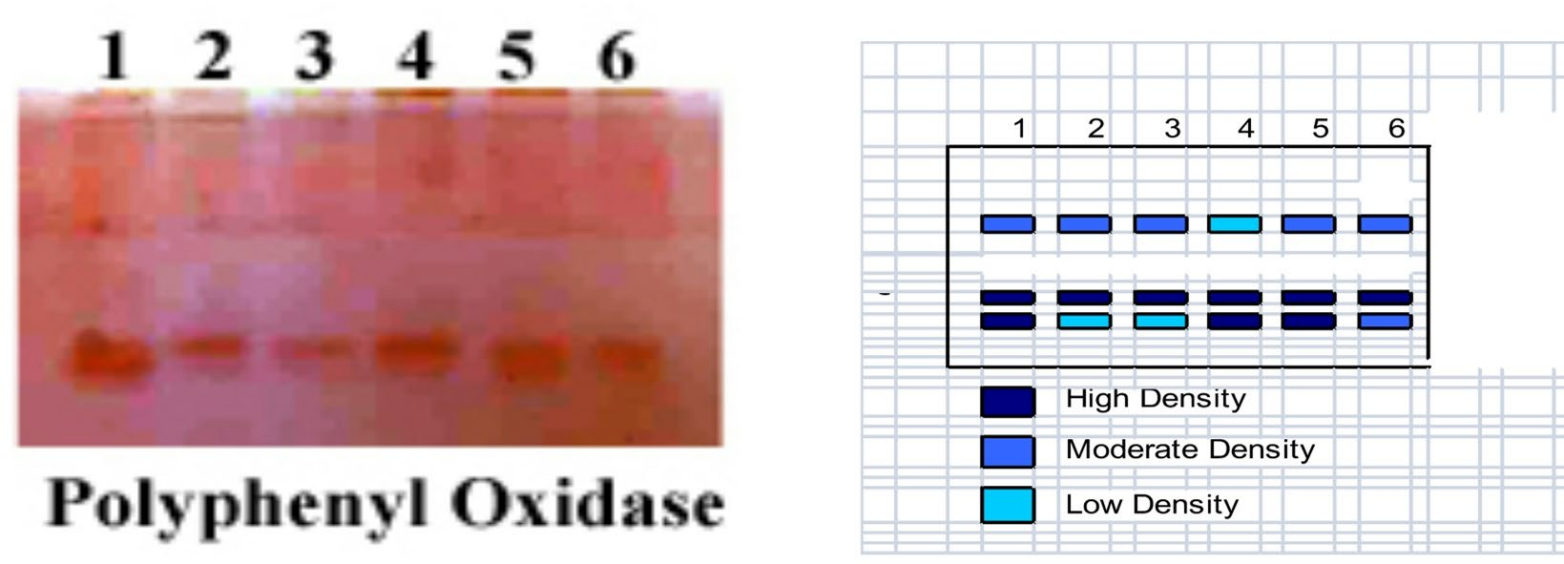
Effect of Heavy metal stress and application of biological composite nanoparticles and their interactions on (**A**) Polyphenol oxidase isozyme and (B) Ideogram analysis of Polyphenol oxidase isozyme of pea plants.

The findings in Fig. 5 demonstrated that the relative mobility and density of the Peroxidase polypeptide bands varied by RF 0.75 and 0.80 in pea. Polymorphism in heavy metal and/or Cu/Zn nanocomposite treatment and/or control, as well as Cu/Zn nanocomposite and heavy metals treated plants, gave 2 bands of POD isozymes POX1: POD2 (0.75 and 0.8) with different densities ranging between low, moderate, and high. Also, it is well shown that several bands for polyphenol oxidase isozymes varied in their densities and mobility according to Cu/Zn nanocomposite and heavy metals concentrations. Additionally, the findings in Fig. 6 demonstrated that the pea plant had differences in the density and relative mobility of the polypeptide bands of Polyphenol Oxidase by RF 0.35, 0.75 and 0.80 treatment with heavy metals and a Cu/Zn nanocomposite to induce polymorphism, where control as well as Cu/Zn nanocomposite and heavy metals treated plants gave 3 bands of PPO isozymes PPO1: PPO3 (0.35, 0.75, and 0.8). It was noted that at 0.75 relative mobility, all bands appeared with high density in all treatments (Table 6).

**Table 6:** Effect of Heavy metal stress and application of biological composite nanoparticles and their interactions on (A) Polyphenol oxidase isozyme and (B) Ideogram analysis of Polyphenol oxidase isozyme of pea plants.

Polyphenyl Oxidase groups	Relative Mobility	1	2	3	4	5	6
PPO1	0.35	1^+^	1^+^	1^+^	1^−^	1^+^	1^+^
PPO2	0.75	1^++^	1^++^	1^++^	1^++^	1^++^	1^++^
PPO3	0.8	1^++^	1^−^	1^−^	1^++^	1^++^	1^+^

++ High density Band, +Moderate density Band, - Low density Band: 1 Present Band, 0 Absent Band.

### Protein profile

**Fig. 7:**
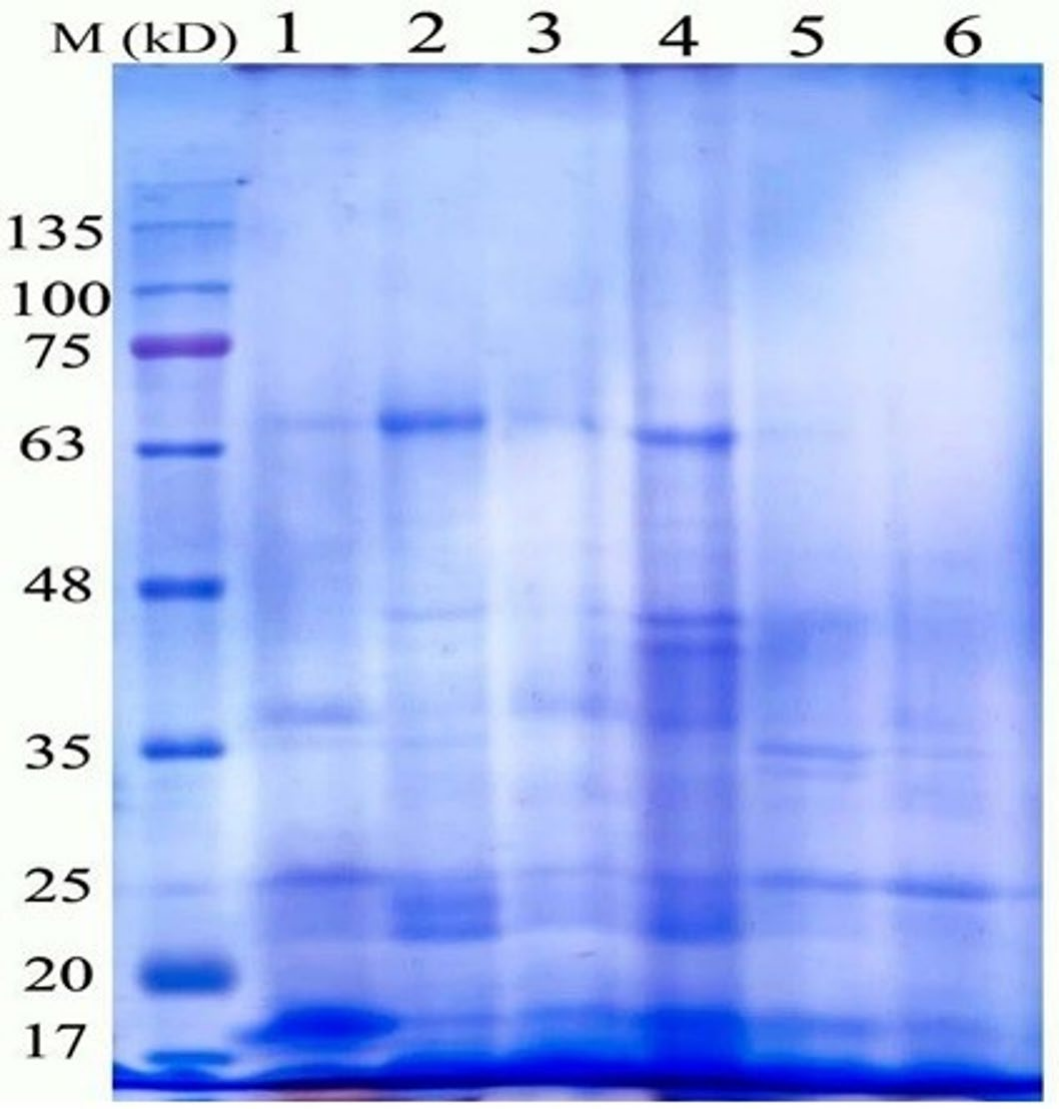
Effect of Heavy metal stress and application of biological composite nanoparticles and their interactions on Protein electrophoretic banding patterns of Pea shoots L1 = Control, L2 = Composite Nano 50 mg L^− 1^, L3 = Composite Nano mg L^− 1^, L4 = Heavy Metal (HM), L5 = Composite Nano 50 + HM, L6 = Composite Nano 100 + HM.

Figure 7; Table 7 show the electrophoretic patterns of pea treated by fresh water, treated by heavy metals, treated by Cu/Zn nanocomposite, and treated with a mixture of heavy metals and Cu/Zn nanocomposite, in turn. These alterations were shown by the appearance and disappearance of certain polypeptides. According to this study, polypeptides with molecular weights of 66 kDa were present in pea plants treated with a Cu/Zn nanocomposite and vanished when heavy metals were added. On the other hand, protein bands with molecular weights of 42 KDa emerged in plants treated with heavy metals and vanished in plants treated with control and nanocomposite. It was noted that protein bands with molecular weights of 25, 23, and 18 appeared in all treatments. The polypeptide band with a molecular weight of 35 kDa was specific only to pea plants treated by Cu/Zn composite Nano 50 HM and Cu/Zn composite Nano 100 HM. The largest number of bands (8 bands) appeared in Cu/Zn composite Nano 100 HM-treated plants. Also, the polypeptide band with molecular weight 51 kDa was specific only to pea plants treated with fresh water only (Control (FW.) and disappeared in all pea plants treated with Cu/Zn nanocomposite alone or heavy metals alone or in combination with Cu/Zn nanocomposite and heavy metals.

**Table 7:** Protein electrophoretic banding patterns of pea plant under the impact of biological composite nanoparticles and Heavy metal stress. L1 = Control, L2 = Composite Nano mg L^− 1^, L3 = Composite Nano 100 mg L^− 1^, L4 = Heavy Metal (HM), L5 = Composite Nano 50 + HM, L6 = Composite Nano 100 + HM.

Band No	M.W bp	Treatments						
		L1	L2	L3	L4	L5	L6	Polymorphism
1	66	+	+	+	-	-	-	Polymorphic
2	51	+	-	-	-	-	-	Unique
3	44	+	+	+	+	+	+	Monomorphic
4	42	-	-	-	+	+	+	Monomorphic
5	38	+	+	+	+	-	+	Monomorphic
6	35	-	-	-	-	+	+	Monomorphic
7	32	-	+	+	+	-	+	Monomorphic
8	25	+	+	+	+	+	+	Monomorphic
9	23	+	+	+	+	+	+	Monomorphic
10	18	+	+	+	+	+	+	Monomorphic

## Discussion

Green nanotechnology has emerged as a compelling and eco-friendly alternative to conventional nanoparticle synthesis methods^52,53^. Traditional approaches often rely on hazardous chemicals, high energy inputs, and non-renewable solvents, which pose serious risks to both human health and the environment^54^. In contrast, green synthesis techniques leverage biological resources such as plant extracts, microorganisms, and biopolymers, which are naturally rich in reducing and stabilizing agents^1,55,56^. These biomolecules — including flavonoids, alkaloids, terpenoids, and proteins — facilitate the formation of nanoparticles under ambient or near-ambient conditions, eliminating the need for toxic reagents or extreme reaction environments. As a result, green nanotechnology offers enhanced biocompatibility, scalability, and environmental sustainability, making it highly attractive for applications in medicine, agriculture, environmental remediation, and beyond^57–59^. The successful biosynthesis of Cu/Zn nanocomposites using *Cakile maritima* Scop extract demonstrates a significant advancement in sustainable nanomaterial production.

The FTIR spectrum displayed prominent absorption bands at 3244.9 cm⁻¹, 2411.7 cm⁻¹, 2164.2 cm⁻¹, 1987.2 cm⁻¹, 1561 cm⁻¹, 1399.8 cm⁻¹, 1053.7 cm⁻¹, 760.3 cm⁻¹, 508 cm⁻¹, and 465 cm⁻¹. These peaks suggest the presence of bioactive compounds such as phenols, alcohols, amines, and proteins, which are commonly found in plant extracts and are known to play essential roles in nanoparticle synthesis. The broad band at 3244.9 cm⁻¹ corresponds to O–H stretching vibrations of hydroxyl groups, typically associated with phenolic compounds and alcohols. Similar broad O–H stretching peaks have been reported in the FTIR spectra of green-synthesized ZnO and CuO nanoparticles, confirming the role of polyphenols in reduction and stabilization processes^60^. The bands at 1561 cm⁻¹ and 1399.8 cm⁻¹ are attributed to N–H bending and C = C stretching vibrations, indicating the presence of amide groups from proteins and aromatic rings from secondary metabolites—features also observed in plant-mediated nanoparticle synthesis by^61^. The absorption band at 1053.7 cm⁻¹ can be ascribed to C–O stretching vibrations of alcohols or carboxylic acids, similar to observations made by^62^, who reported this region as indicative of flavonoids and sugars involved in nanoparticle stabilization. The peaks at lower wavenumbers—particularly between 465 and 760.3 cm⁻¹—are characteristic of metal–oxygen (M–O) stretching vibrations, which confirm the formation of metal oxide bonds, including Cu–O and Zn–O, as also reported by^30^ in Cu/ZnO nanocomposites. These findings are consistent with multiple reports on plant extract-mediated synthesis of metal and metal oxide nanoparticles, where functional groups originating from biomolecules serve as natural reducing and stabilizing agents^63^. Therefore, the observed FTIR peaks not only confirm the successful formation of the Cu/Zn nanocomposite but also highlight the essential role of phytochemicals in controlling nanoparticle morphology and stability during green synthesis.

The XRD pattern of the synthesized Cu/Zn nanocomposite reveals sharp and well-defined peaks at 2θ values around 31.9°, 34.2°, 36.4°, 47.9°, 56.9°, 62.8°, and 67.9°, indicating a crystalline nature consistent with previously reported CuO and ZnO phases. These peaks closely match the standard diffraction patterns of monoclinic CuO and hexagonal wurtzite ZnO, as documented in the JCPDS cards No. 80-1917 for CuO and No. 36-1451 for ZnO. These results are in close agreement with previous studies. For instance^64^, synthesized Cu-doped ZnO nanoparticles using *Aloe vera*and reported diffraction peaks at 2θ ≈ 31.7°, 34.4°, and 36.2°, which also match the wurtzite phase of ZnO, with slight shifts attributed to Cu incorporation. Moreover^65^, reported that the introduction of Cu into ZnO caused minor peak broadening and slight shifts in 2θ values, indicating lattice distortion due to ionic substitution of Zn²⁺ by Cu²⁺. This is consistent with the current findings, suggesting that Cu ions are either doped into the ZnO lattice. The similarity between the diffraction patterns observed in this study and those reported in the literature confirms that green synthesis methods can effectively produce crystalline Cu/Zn nanocomposites, with structural features comparable to those obtained by conventional or chemical synthesis routes. Transmission Electron Microscopy (TEM) analysis revealed that the Cu/Zn nanocomposites exhibit a predominantly spherical morphology with slight particle aggregation. Individual nanoparticles measured directly from the micrograph were found to range between 65 and 90 nm in diameter. This morphology is indicative of successful nanoparticle formation and suggests effective stabilization, likely facilitated by bioactive compounds present in the plant extract. Similar spherical morphologies have been widely reported in green synthesis studies. For example^66^, observed nearly spherical Cu/ZnO nanoparticles with slight agglomeration when synthesized using *Sambucus nigra*extract, with particle sizes ranging from 10 to 100 nm. Likewise^67^, reported spherical CuO-ZnO nanostructures synthesized via a plant-mediated method, with comparable size distributions and aggregation behavior attributed to natural capping agents. Complementary energy-dispersive X-ray spectroscopy (EDX) analysis (Fig. 3b) confirmed the elemental composition of the nanocomposites, showing distinct peaks corresponding to carbon (C), oxygen (O), copper (Cu), and zinc (Zn). The dominant signals for copper and zinc confirm their substantial incorporation into the nanocomposite structure. The detection of carbon and oxygen is consistent with the presence of organic molecules from the plant extract, which may function as reducing and stabilizing agents—a finding supported by^67^, who noted similar elemental profiles in EDX spectra of green-synthesized metal oxide nanoparticles. Additionally^68^, reported that Cu/ZnO nanocomposites prepared using *Annona glabra* extract also showed strong Cu and Zn peaks alongside C and O, confirming the dual role of the extract in both metal reduction and surface functionalization. The observed morphology and elemental composition in this study are consistent with previous reports on plant-mediated synthesis of Cu/Zn nanocomposites, reinforcing the reliability and reproducibility of green synthesis as a sustainable approach to nanomaterial fabrication.

Increased concentrations of heavy metal pollutants in arable regions negatively impact agricultural plants’ physiochemical activities, growth, yield, and biomass production^25,69^. The development of plants is inhibited by heavy metal stress because it reduces root formation and growth, which in turn reduces water and nutrient intake and translocation^70^.

Copper and zinc nanoparticles are highly regarded in agriculture due to their numerous applications, including nano fertilizers, nanopesticides, nanosensors, nano-zeolites, and antioxidants for better protection of crops^71^. When present in optimal concentrations, copper and zinc, two vital micronutrients, support plant growth and development by controlling a number of physiological and biochemical functions. According to^72^, copper is essential for respiration, photosynthesis, protein and lignin production, cell wall metabolism, and glucose metabolism. Zinc is involved in the creation of proteins, auxins, nucleic acids, chlorophyll, cell division, cell growth, and carbohydrates^73^. Because both metals may gain or lose electrons, they can function as cofactors for a number of enzymes that control significant plant biological processes^74^. The use of copper and zinc nanoforms as nanofertilizers has great promise for improving plant development and stress tolerance^75,76^.

Because of their high conductivity, non-toxicity, easy availability, eco-friendliness, affordability, and inert nature, copper oxide and zinc oxide nanoparticles have been chosen over other types of nanoparticles^71,77,78^ Exogenous application of CuO NPs. According to current research, it has improved cadmium remediation in Phaseolus vulgaris^79^, induce tolerance to salinity stress in *Solanum lycopersicum*^80^, and increase photosynthetic efficiency and antioxidant activity in Brassica juncea^81^. Numerous plants, notably *Trigonella foenum-graecum*, have been shown to benefit from ZnO NPs’ ability to reduce salt stress *Solanum lycopersicum*^82^, *Vicia faba*^83^, *Brassica napus*^84^ and *Sorghum bicolor*^85^.

Because it promotes the targeted entry of nutrients into the plant system with high nutrient usage efficiency, foliar application of nanoparticles produces faster and better outcomes than soil application^86^. Previous studies have extensively documented the use of foliar application of CuO/ZnO/SiO_2_NPs) nanoparticles to promote plant development in stressful environments and mitigate the adverse effects of salt stress^83,87^. Nevertheless, the effects of nanoparticles on plants differ depending on the kind of plant, stage of development, size, concentration, and physicochemical characteristics of the nanoparticles^88^. According to^89^. Metal stress inhibits photosystem II (PSII), disrupts the electron transport chain, reduces chlorophyll production, and stops the Calvin cycle.

Our results concur with those of^90^ and^91^, who found that metal-stressed *Oryza sativa* and *Lolium perenne* plants had decreased biosynthesis, respectively. This might be because HMs decrease the absorption of carbon dioxide (CO2) by either interacting with the thiol group of RUBISCO, an enzyme involved in CO_2_ fixing, or by blocking the action of RUBP carboxylase. By inducing ethylene synthesis, which in turn stimulates the jasmonic acid signaling pathway, HMs also cause senescence in plants^92^.

The rate of photosynthesis is reduced in stressed plants due to a reduction in the biosynthesis of photosynthetic components^93^. Pb stress most likely reduced the activity of the enzymes that synthesize chlorophyll. In plants under pb stress, the increased production of ROS may have harmed the structure of the chloroplasts and the chlorophyll protein complex, while also increasing the activity of chlorophyllase. The deleterious effects of As-induced stress on the electron transport chain, photosynthesis content, photosystem II, gas exchange parameters, and Calvin cycle were also demonstrated by^94^.

*Pisum sativum* plants grown in Pb-contaminated substrate may have less photosynthesis because of the substitution of Pb with vital mineral elements linked to the formation of photosynthetic pigments. However, the production of photosynthetic pigments was enhanced, and the toxicity of lead was much reduced when plants treated with Cu/Zn nanocomposite had an improvement in their antioxidant system. Heavy metals can directly hinder photosynthesis in plants in several ways, including by blocking the chlorophyll biosynthesis pathway^95,96^, decreasing chlorophyll content by increasing chlorophyllase activity (Kapoor et al., 2019), and altering the ultrastructure of the chloroplast^95,97^.

Pb causes plants to produce too many reactive oxygen species (ROS) and peroxidize their membrane lipids^98^. This causes oxidative stress by changing the quantity of non-enzymatic antioxidants and the activity of antioxidant enzymes^99^. Additionally, Pb decreases photosystem II (PSII) activity, chlorophyll content, and cell membrane permeability, which results in metabolic problems^100^.

Biological Cu/Zn nanocomposite treatment, on the other hand, led to a notable increase in photosynthetic pigments in both the heavy metal-treated and control pea plants. As indicated by^101^. The present study’s findings are consistent with the beneficial effects of CuO NPs on *T. aestivum* plants’ gas exchange characteristics, photosynthetic metrics, and chlorophyll concentrations.

Fouda et al.^2^ corroborated our findings by stating that the decrease in chlorophyll content may be restored to a level comparable to the control by applying CuNPs or CubNPs + Cu topically. The notable increase in photosynthetic pigments due to the application of the Cu-Zn nanocomposite may indicate that the nanocomposite helps maintain the structural integrity of chloroplasts and photosystems, ensuring efficient energy production even under stress conditions^102,103^.

To preserve chlorophyll content under stressful conditions, carotenoids in plants function as an antioxidant to shield it from oxidative stress^104^. Their findings demonstrated that, in comparison to water-treated plants, nano-Cuo priming enhanced the carotenoid content of maize under drought, which may aid plants in fending off the process of chlorophyll breakdown and preserving greater chlorophyll concentrations. Thus, higher carotenoid content influenced by the application of Cu/Zn nanocomposite helped to improve pea heavy metal tolerance in this study. When compared to control (FW), pea plants treated with Cu/Zn nanocomposite exhibited significantly higher levels of total soluble carbohydrates in both the shoot and the root. This could be because copper plays a key role in respiration, photosynthesis, the metabolism of carbohydrates and cell walls, as well as the synthesis of proteins and lignin^72^.

Photosynthetic pigments and Carotenoids are affected by HM contents, which also raise MDA levels. They also damage the structure of chloroplasts by closing their stomata^105,106^ and alter the plant’s metabolic processes, electron transport chain (ETC), and chloroplasts^107^. According to^108^, ZnO nanoparticles have been demonstrated to raise the protein content of cabbage, which is followed by a rise in chlorophyll and carotenoids.

According to^109^, wheat plants treated with CuO/ZnO NPs also showed increased growth, which could be because CuO NPs are less soluble. When combined, these results showed that applying Zn-based NPs improved plant resistance to stress. Singh et al.^110^, Cu is also involved in several enzyme systems that control the pace of several biochemical reactions in plants. Additionally, it is necessary for photosynthesis, which helps plants metabolize proteins and carbohydrates and is necessary for plant respiration^110–113^.

According to^73^ zinc is involved in the creation of proteins, auxins, nucleic acids, chlorophyll, cell division, cell growth, and carbohydrates. Both metals can function as cofactors for several enzymes that control significant metabolic activities in plants because of their capacity to acquire or lose electrons^74^. Plant development and resistance to environmental stress can be significantly improved by using copper and zinc nanoforms as nanofertilizers^75,76^. Because of their high conductivity, non-toxicity, easy availability, eco-friendliness, affordability, and inert nature, (CuO) and (ZnO) nanoparticles have been chosen over other types of nanoparticles^71,77^.

Our study’s higher carbohydrate content may be because zinc oxide nanoparticles are a very efficient source of zinc, which is necessary for normal plant growth and development, because they aid in the synthesis of auxins, which in turn improve plant cell wall development and cell differentiation, as well as the metabolism of proteins and carbohydrates^19^. Our research results demonstrated also that heavy metal treatment considerably reduced the amount of total soluble proteins in the shoots of pea plants. This might be as a result of Pb lowering the amounts of sugar and amino acids that support cell turgidity and cell wall flexibility^114^. When Cu/Zn nanocomposite was applied, however, the amount of total soluble protein increased in comparison to control plants. This increase may refer to that ZnO NPs can reduce lipid peroxidation and raise total soluble protein and photosynthetic pigment levels, according to^107^ study. Increasing the amount of protein (TSP) and total soluble sugar (TSS) has a role in pea plant stress tolerance, energy generation, and osmoregulation. By regulating osmotic potential and membrane and biomolecule breakdown, plants stabilize the levels of protein and sugar.

Our finding demonstrates that application of Cu/Zn nanoparticles significantly increases the total phenol contents of pea plants under lead (Pb) stress. By directly enhancing nutrition intake, water molecule conduction from roots to higher sections of the plant, and vascular bundle tissue cells, zinc and copper ions improved the integrity of cell membranes^115^. Pea plants under Lead stress had a larger phenolic content, according to our results in Table 4. Pb poisoning may have enhanced the bioactivity of phenylalanine ammonia-lyase, a key enzyme in the phenylalanine pathway that produces flavonoids and phenolic chemicals. Bhadwal et al.^116^, also found that metal-stressed *Oryza sativa* plants produce more flavonoids and phenolic chemicals, which reduce stress^117^. *Tithonia diversifolia* plants have been shown to have high endogenous concentrations of flavonoids and phenols following exposure to Pb-acetate, suggesting that these compounds play a crucial role in preventing Pb toxicity^118^. Various osmolytes, such as proteins and sugars, as well as secondary metabolic compounds, like phenolic compounds, effectively reduce active free radicals and protect plant cells from the harmful effects of Pb-induced oxidative stress. They also play a defensive role in the damage caused by Pb toxicity^119^. Following this pattern^120^, discovered that applying 50 ppm of copper topically to onion plants greatly improved their yield, quality, and nutrient uptake as measured by the fresh and dry weights of the bulbs, their diameter, their neck diameter, their height, and the percentages of nitrogen, phosphorus, potassium, copper, and protein in the bulbs at harvest time when compared to the untreated plant. Furthermore, at the cellular level of plants, copper contributes to oxidative phosphorylation, iron mobilization, transcription signaling, and protein trafficking machinery^121–123^.

The generation of reactive oxygen species (ROS) is the main defensive mechanism of plants against abiotic and biotic stresses^25,124^. The antioxidant system and electron transport chains are adversely affected when HM stress results in stomatal closure and elevated ROS generation. Lipid peroxidation brought on by ROS generation may further impair the integrity and functionality of cell membranes. Das and Roychoudhury^125^, Emamverdian^126^, Hoque^127^, Kärkönen^128^. According to our data, pea plants treated with heavy metals like lead acetate showed a considerable rise in H_2_O_2_ and MDA buildup. This might be because Pb ions can also lower saturated fatty acids and raise unsaturated fatty acids in the membranes of many plants, which can lead to lipid peroxidation in some situations^119^. According to^103^, CuO NPs increase the activity of the enzymes SOD, POD, and CAT in *T. aestivum* plants while decreasing the concentrations of EL, MDA, and H_2_O_2_.

MDA and H_2_O_2_ concentrations may be changed by nanoparticles (NPs), which reduces the buildup of both substances in pea plants. The effectiveness of the Cu/Zn nanocomposite in strengthening the defensive mechanism of the *Pisum sativum* plant was highlighted by the current findings. ZnO NPs have been shown in studies on *O. sativa* to reduce ROS molecules, including H_2_O_2_, and may maintain plant membrane oxidation by reducing MDA levels^129^.

The results of our latest study on peas were the same. One possible explanation for the decrease in MDA levels and increased photosynthetic activity might be an enhanced antioxidant system that preserves membrane integrity by lowering MDA production and boosting antioxidative activity. When plants are under stress, ROS create hydroxyl radicals, lipid hydrogen peroxide through a Fenton-like reaction, and aldehydes (malondialdehyde). Activating gene expression, cell cycle, and protein transcription, H_2_O_2_ concentration in plants interacts with thiol-containing proteins and creates a variety of signaling pathways^111,130^.

Pea plants under Lead stress had a larger phenolic content, according to our results in Table 4. Pb poisoning may have enhanced the bioactivity of phenylalanine ammonia-lyase, a key enzyme in the phenylalanine pathway that produces flavonoids and phenolic chemicals. Bhadwal et al.^116^, also found that metal-stressed *Oryza sativa* plants produce more flavonoids and phenolic chemicals, which reduce stress^117^. *Tithonia diversifolia* plants have been shown to have high endogenous concentrations of flavonoids and phenols following exposure to Pb-acetate, suggesting that these compounds play a crucial role in preventing Pb toxicity^118^.

Likewise, anthocyanins and phenols, and flavonols—all of which have antioxidant properties—were more abundant in *Hippophae rhamnoides* cultivated in Pb-affected soil^131^. Wiszniewska^132^, found that exogenous phenolic acids increased antioxidant activity against Pb-stress, which led to tolerance in *Daphne jasmine*. According to reports, phenolic compounds can function as radical scavengers or radical-chain breakers^133^.

Consequently, phenolic compounds—considered the most important antioxidative plant components^134^ for prevention and recuperation from heavy metal damage^135^—accumulated in greater amounts in stressed plants. These compounds’ carboxyl and hydroxyl groups, which may chelate with other metallic ions, give them their anti-oxidative properties^136^. Chelators, which are substances present in the root exudates of plants that can bind to harmful metal ions, are used to limit the uptake of heavy metals by plants and specific plant organs. Chelating substances include organic acids, amino acids, and phenolic chemicals^137–140^.

Similar outcomes were noted in potato plants, where ZnO-NP treatment increased the overall content of phenolic compounds. At doses of 100, 300, and 500 ppm, the increase was 1%, 20%, and 22%, respectively^141,142^. The primary impact of NPs on phenolic compounds, since these chemicals are crucial for plant productivity and adaptability to biotic and abiotic stressors^143^.

Because of their redox properties, phenolic compounds can directly destroy active oxygen molecular species in addition to acting as metal chelators under heavy metal stress. According to study of^144^ these characteristics may be essential for the breakdown of peroxides, the extinction of singlet and triplet oxygen, and the absorption and neutralization of free radicals. Therefore, the main cause of the increases in antioxidant activity of plants exposed to NPs is phenolic compounds, which are strong ROS scavengers and may also inhibit enzymes that produce free radicals^145^. However, its molecular structure is mostly responsible for its antioxidant effect^141^.

According to^2^. The beneficial impacts of manufactured copper nanoparticles have been demonstrated to increase all growth parameters, photosynthetic pigments, proline, phenol, anthocyanin, shikimic acid, and enzyme activities in comparison to the control. These findings corroborate with our findings. (Pb) is known to negatively affect a plant’s growth, morphology, and photosynthetic processes. Furthermore, when too many very harmful ROS accumulate, Pb stress causes plants to oxidatively damage proteins, lipids, and nucleic acids^146^.

By decreasing the activity of aminolaevulinic acid dehydratase (ALAD), Pb also affects photosynthesis^147^. Table 8’s data demonstrated that using a Cu/Zn nanocomposite significantly reduced the yield of Pb buildup. These findings are consistent with research that found ZnO NPs also lessened HM stress by reducing HM absorption by plants, shielding them from HM toxicity^148^. The symptoms of oxidative stress caused by Cd and Pb toxicity can be reduced by treatment with ZnO NPs^7^.

One of the main features of ZnO NPs is their ability to eliminate and degrade pollutants from the air and water. Using HMs adsorption and strong physical and chemical characteristics, ZnO NPs may be transformed into a single, distinct NP^149^. Our findings support research that found ZnO NPs protect Leucaena leucocephala from oxidative stress brought on by Cd and Pb, hence mitigating the harmful effects of these heavy metals^150^.

**Table 8:** Effect of Heavy metal stress and application of biological composite nanoparticles and their interactions on (A) Peroxidase isozyme and (B) Ideogram analysis of peroxidase isozyme of pea plants.

**Pox2**	0.8	1^++^	1^+^	1^+^	1^++^	1^++^	1^+^

++ High density Band, +Moderate density Band, - Low density Band; 1 Present Band, 0 Absent Band.

Our findings also concur with research that found that using nanoparticles to remove harmful heavy metals from wastewater is an effective method. This work used nanoparticles (Fe3O4, ZnO, and CuO) as new sorbents to explore the removal of Cd2+, Cu2+, Ni2+, and Pb + 2^151^. One of the most crucial remediation techniques for the removal of lead is phytoextraction^152^, and it has been shown that adding NPs to plants can improve Pb phytoextraction. According to research, 30% of Pb was removed from the soil after one month and 44.39% after three months when Lolium (ryegrass) and nanohydroxyapatite were applied together^150,153^.

These results demonstrate how well the CuO/ZnO nanocomposite reduces lead levels in different plant sections and absorption, which may benefit Pisum sativum plants’ general health and the safety of agricultural goods. On the other hand, using CuO and ZnO NPs together may improve pea productivity and growth in two ways. This may be because the Cu/Zn nanocomposite has a beneficial effect on lowering the amount of soluble heavy metals (HMs) like Pb in the soil. For many enzymes involved in plant metabolism, ZnO NPs function as a cofactor^154^.

Additionally, it was recently revealed that genes encoding antioxidant enzymes are upregulated in hydroponic cultures when zinc oxide nanoparticle concentrations are greater, such as 50 and 100 mg/L^155^. According to^156^, ZnO NPs improve chlorophyll content and plant biomass under metal toxicity by boosting antioxidant activity, decreasing ROS compounds, and limiting translocation and accumulation factors in bamboo plant organs. This strengthens bamboo’s tolerance to HM stress. Furthermore, tomato plants exposed to nanocarbons may produce genes associated with stress^157^ that elicit antioxidant activity, including POX, which might enhance stressed plants’ growth and development^158^.

According to^129^, Zn NPs can thereby boost the activity of antioxidant enzymes such as SOD, POD, and CAT in plants that have been impacted by heavy metals. This is the case in O. sativa^129^ under Cd stress. However, through the stimulation of proline biosynthesis gene expression, Zn NPs can enhance proline buildup under hazardous situations^129^. One of the most important defensive mechanisms to deal with plant abiotic stress is the antioxidant enzyme activity that initiates the scavenging of ROS molecules. In^159^, when ZnO NPs were sprayed on maize leaves, there was a decrease in both Cd absorption and Cd-induced oxidative stress.

ZnO NPs increased plant growth, photosynthetic index, and chlorophyll content while decreasing Na concentration in sunflower leaves^160^. Results from treating wheat plants with ZnO NP have been similar. ZnO NPs can mediate the increase in photosynthetic pigments and the concomitant decrease in lipid peroxidation in soil-grown Coriandrum sativum plants, according to^161,162^ studies. Consequently, ZnO-mediated NPs enhance photosynthetic pigments by stopping the production of ROS, which may aid plants in surviving harsh conditions. Dimkpa^163^, Dimkpa^9^, shows that ZnO, boric oxide, and CuO NPs work together to lessen drought stress in G. max. As a result, plants treated to CuO or ZnO NPs showed cross-protection against a range of hazards, including metal and drought.

POD enzymes acquire electrons to dismutase the H_2_O_2_ level in plants during HM stress. Zinc oxide nanoparticles’ low concentration was converted into zinc ions, which are vital cofactors for enzymes involved in metabolism and micronutrients for plant development. According to^164^. Pigeon pea under stress has been shown to have elevated superoxidase and peroxidase activity. According to research of^165^. Rice, soybean, and *Athium wardii* seedlings all displayed higher POD contents when exposed to Pb stress^111^.

Furthermore, the application of Cu/Zn composite considerably increased the activity of peroxidase and polyphenol oxidase in pea plants compared to plants treated with lead acetate alone in both the lead acetate-stressed and control groups watered with distilled water. According to^166^ plants’ antioxidant defense systems also incorporate enzymatic compounds like POD and PPO that eliminate ROS. To detoxify metal-trigger ROS, higher plant species activate non-enzymatic antioxidants such as phenolic compounds and enzymatic antioxidant systems like SOD, POD, and CAT^167^.

Nguyen^168^, observed that nano-CuO priming increases the anthocyanin content and the activity of the enzyme’s ascorbate peroxidase and superoxide dismutase during drought stress. Our results are in line with their findings. This may Pb to a decrease in excessive reactive oxygen species production and, consequently, higher adaptation to drought stress conditions in maize. By using nano-CuO to increase plant biomass and maize production, these discoveries may find practical applications in agriculture. At 10 and 100 mg L − 1 of CuO NPs, respectively, our results are comparable to those of Costa and Sharma, who employed CuO NPs in Oryza sativa and reported elevated expression of enzyme antioxidants, APX and SOD, which likewise shield plants from oxidative stress^81,169^. Under heavy metal stress conditions, the current study showed that increased activity of POX and PPO enzymes, which aid plants in detoxifying excess ROS, led to decreased ROS buildup in plants treated with Cu/Zn nanocomposite. Prior research, which found that sprayed copper compound nanoparticles enhanced antioxidant systems, including SOD and peroxidase activities, as well as total antioxidant levels, corroborated these findings^110,170,171^.

Several regulatory proteins have their redox state directly changed by signaling ROS, which also alters transcription and translation. This sets off an acclimation response that reduces the quantity of ROS produced by metabolism and the effect of plant stress on metabolism^172,173^. Furthermore, the application of Cu/Zn nanocomposite boosted the amount of phenolics in pea under heavy metal stress, indicating that it not only influences the activity of ROS-scavenging enzymes but also increases antioxidant production in pea in response to heavy metal stress. In response to heavy metal stress, the application of Cu/Zn nanocomposite in pea enhanced the activity of ROS-scavenging enzymes and antioxidants to detoxify more ROS molecules. This resulted in the preservation of chlorophyll pigments, which may ultimately preserve pea grain production. By altering transcriptional factors, post-translational modification, and stress-inducible gene expression, the CuO/ZnO nanocomposite also controls the molecular process.

We try to investigate the molecular processes behind peas’ resilience to Pb stress. These findings will give the scientific foundation for choosing and producing Pb-resistant pea varieties as well as a fundamental theoretical underpinning for advancing our understanding of pea genetic breeding. The response of plants to Pb stress, such as *Zea mays* L. Li^174^, and *Vernicia fordii*^175^, has been better understood in recent years thanks to integrated transcriptomics and metabolomics investigations.

Because omics technology is still being developed and used, transcriptome analysis is a new experimental technique for examining how crop genomes and their physiological regulatory systems operate for stress tolerance. With transcriptome analysis based on RNA sequencing (RNA-seq), gene expression patterns and molecular processes of plant growth and development, as well as the response to abiotic stress, can be investigated^176^. To understand plant metabolic networks and metabolic regulation, as well as to demonstrate the close relationship between plants and their environment, metabolomics, as opposed to transcriptomics, focuses on detecting and quantifying low molecular weight metabolites in biological samples^177^.

## Conclusion

This research shows that applying green-synthesised Cu–Zn nanocomposites topically to pea (*Pisum sativum* L.) plants efficiently reduces lead (Pb) toxicity. Cu–Zn nanocomposite application, especially at 100 mg L⁻¹, considerably reduced the impacts of Pb stress on photosynthetic pigments, metabolic performance, and antioxidant balance while increasing oxidative damage and Pb accumulation. The nanocomposites decreased hydrogen peroxide and lipid peroxidation while increasing photosynthetic efficiency, osmolyte and protein accumulation, and antioxidant defense. Pb absorption was significantly reduced, and protein expression patterns and antioxidant isozymes were clearly modulated. Together, these results show that green-synthesized Cu–Zn nanocomposites enhance pea plants’ physiological and biochemical resistance to Pb stress and offer a viable strategy for environmentally friendly crop management in polluted soils.

## Supplementary Information

Below is the link to the electronic supplementary material.

Supplementary Material 1

Supplementary Material 2

Supplementary Material 3

## Data Availability

All data and materials available.
