# Author Correction: Foliar application of green-synthesized Cu–Zn nanocomposites: improve physiological responses, isozymes activity, and photosynthetic traits in lead-stressed pea (*Pisum sativum* L.) plants

**DOI:** 10.1038/s41598-026-49797-1

**Published:** 2026-04-23

**Authors:** Mahmoud S. Osman, Salem S. Salem, Hossam M. Fouda, Amr H. Hashem, Sahar I. ELshennawy, Eman N. Mustafa, Eman G. El-Hosary

**Affiliations:** 1 Botany and Microbiology Department, Faculty of Science, Al-Azhar University, Cairo11884, Nasr City, Egypt; 2 Botany and Microbiology Department, Faculty of Science, Girl’s Branch, Al-Azhar University, Cairo, Egypt; 3 Botany and Microbiology Department, Faculty of Science, Damanhour University, Damanhour, Egypt

Correction to: *Scientific Reports* 10.1038/s41598-026-43558-w, published online 26 March 2026

The original version of this Article contained an error in the ‘Author contributions’ section.

As a result, where:

“M.S.O., S.S.S., H.M.F., A.H.H.: Writing – original draft, Methodology, Conceptualization. Ebrahim Saied: Methodology, Formal analysis, Writing – original draft. S.I.E., E.N.M., E.G.E.: Software, Resources, Investigation’s., S.S.S., H.M.F., A.H.H. – review& editing, Writing – original draft, Supervision, Data curation, Conceptualization.”

now reads:

“M.S.O., S.S.S., H.M.F., A.H.H.: Writing – original draft, Methodology, Conceptualization. S.I.E., E.N.M., E.G.E.: Software, Resources, Investigation, S.S.S., H.M.F., A.H.H. – review& editing, Writing – original draft, Supervision, Data curation, Conceptualization.”

The original Article has been corrected.

