# Supplementary figures and images for: Foliar application of green-synthesized Cu–Zn nanocomposites: improve physiological responses, isozymes activity, and photosynthetic traits in lead-stressed pea (Pisum sativum L.) plants
Source: Sci Rep. 2026 Mar 26;16:10487. doi: 10.1038/s41598-026-43558-w (PMC13031724; doi:10.1038/s41598-026-43558-w)

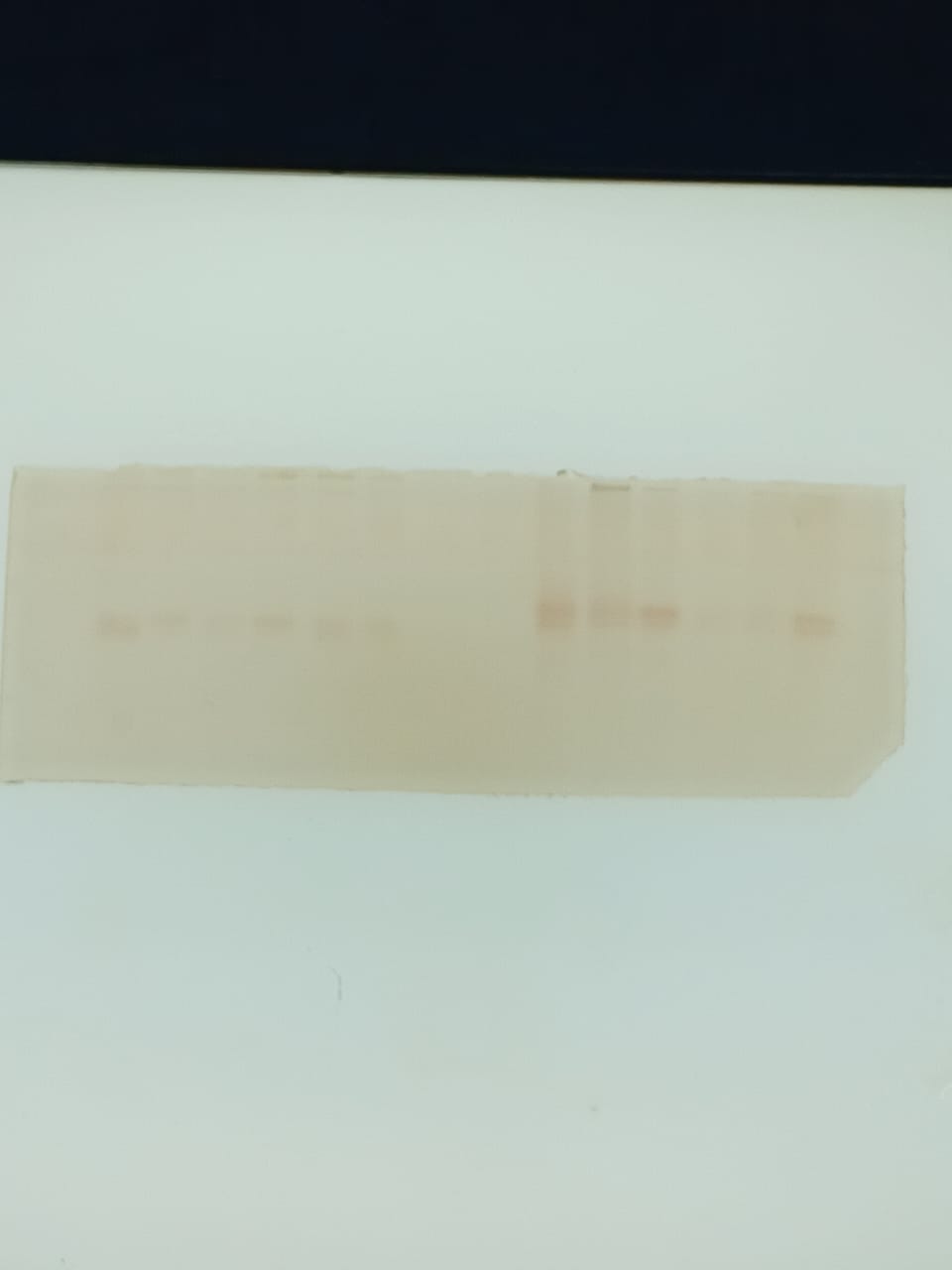

Supplement: Supplementary file 1 — Supplementary Material 1 [file 41598_2026_43558_MOESM1_ESM.jfif]

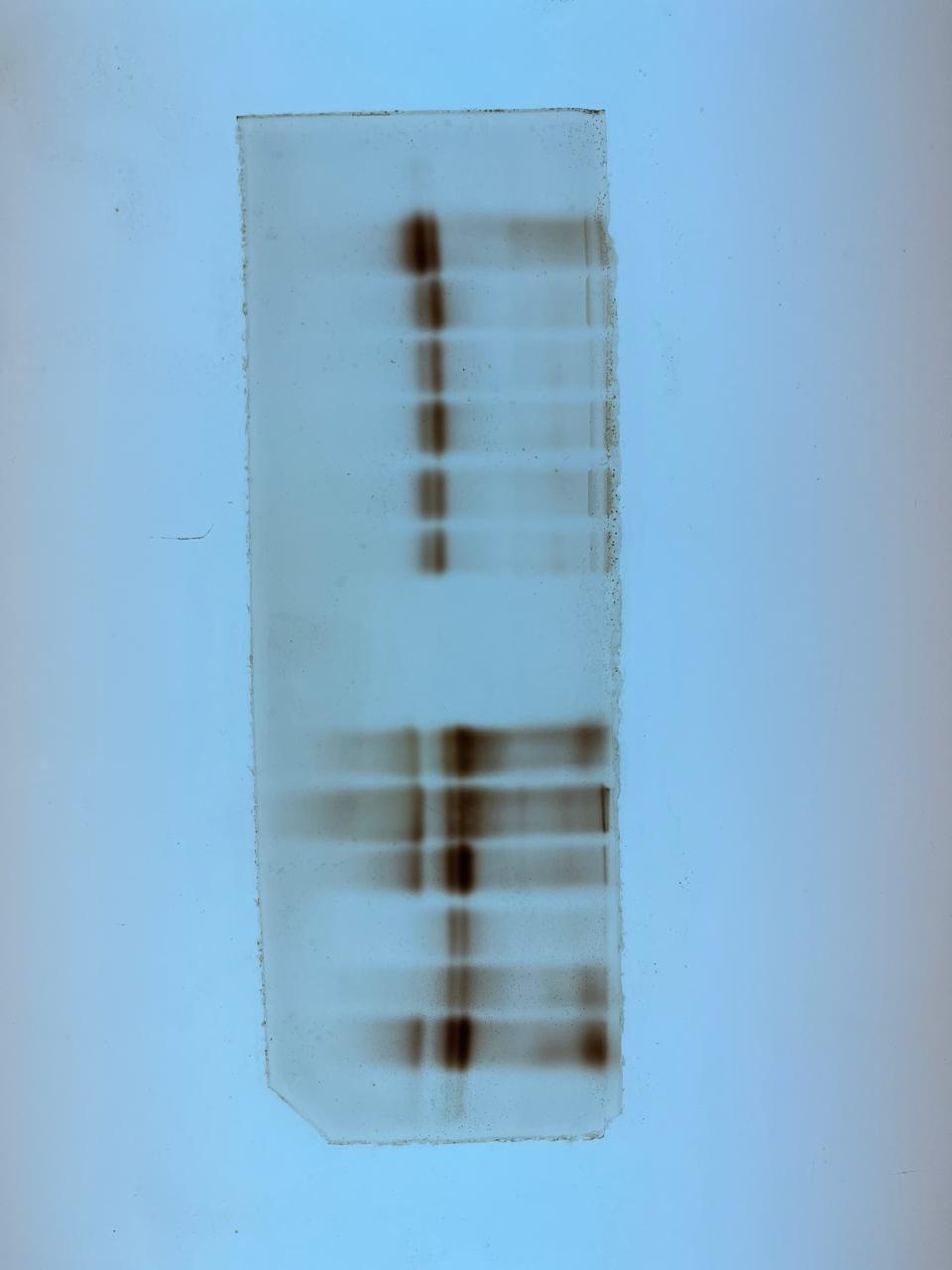

Supplement: Supplementary file 2 — Supplementary Material 2 [file 41598_2026_43558_MOESM2_ESM.jfif]

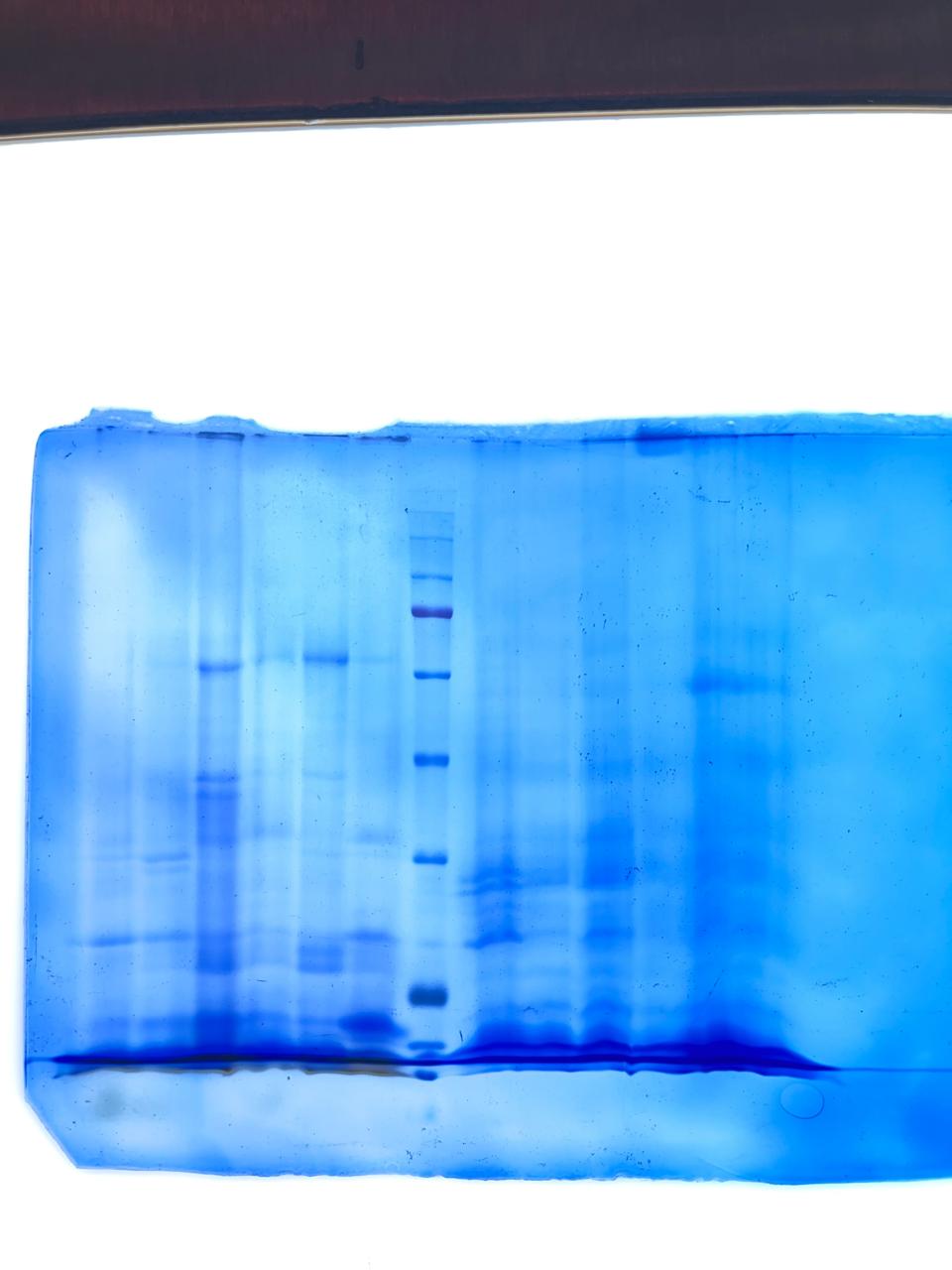

Supplement: Supplementary file 3 — Supplementary Material 3 [file 41598_2026_43558_MOESM3_ESM.jfif]
